# Spectrum of De Novo Cancers and Predictors in Liver Transplantation: Analysis of the Scientific Registry of Transplant Recipients Database

**DOI:** 10.1371/journal.pone.0155179

**Published:** 2016-05-12

**Authors:** Jie Zhou, Zhenhua Hu, Qijun Zhang, Zhiwei Li, Jie Xiang, Sheng Yan, Jian Wu, Min Zhang, Shusen Zheng

**Affiliations:** 1 Division of Hepatobiliary and Pancreatic Surgery, Department of Surgery, First Affiliated Hospital, School of Medicine, Zhejiang University, Key Laboratory of Combined Multi-Organ Transplantation, Ministry of Public Health, Key Laboratory of Organ Transplantation, Hangzhou, Zhejiang, China; 2 State Key Laboratory for Diagnosis and Treatment of Infectious Diseases, Collaborative Innovation Center for Diagnosis and Treatment of Infectious Diseases, The First Affiliated Hospital, School of Medicine, Zhejiang University, Hangzhou, Zhejiang, China; Ohio State University Medical Center, UNITED STATES

## Abstract

**Background:**

De novo malignancies occur after liver transplantation because of immunosuppression and improved long-term survival. But the spectrums and associated risk factors remain unclear.

**Aims:**

To describe the overall pattern of de novo cancers in liver transplant recipients.

**Methods:**

Data from Scientific Registry of Transplant Recipients from October 1987 to December 2009 were analyzed. The spectrum of de novo cancer was analyzed and logistic-regression was used to identify predictors of do novo malignancies.

**Results:**

Among 89,036 liver transplant recipients, 6,834 recipients developed 9,717 post-transplant malignancies. We focused on non-skin malignancies. A total of 3,845 recipients suffered from 4,854 de novo non-skin malignancies, including 1,098 de novo hematological malignancies, 38 donor-related cases, and 3,718 de novo solid-organ malignancies. Liver transplant recipients had more than 11 times elevated cancer risk compared with the general population. The long-term overall survival was better for recipients without de novo cancer. Multivariate analysis indicated that HCV, alcoholic liver disease, autoimmune liver disease, nonalcoholic steatohepatitis, re-transplantation, combined transplantation, hepatocellular carcinoma, immunosuppression regime of cellcept, cyclosporine, sirolimus, steroids and tacrolimus were independent predictors for the development of solid malignancies after liver transplantation.

**Conclusions:**

De novo cancer risk was elevated in liver transplant recipients. Multiple factors including age, gender, underlying liver disease and immunosuppression were associated with the development of de novo cancer. This is useful in guiding recipient selection as well as post-transplant surveillance and prevention.

## Introduction

Liver transplantation is a life-saving therapy for end stage liver disease. The number of transplant cases has remained relatively stable in the United States in recent years, accompanied by improved graft survival and overall survival rates [1, 2]. However, the increased tumor burden due to longer life expectancy and use of immunosuppression regimens for preventing graft rejection, as well as cancer-related virus infection (including hepatitis B virus (HBV), hepatitis C virus (HCV), and Epstein-Barr virus (EBV)), has substantially impaired the overall survival of recipients.

Previous studies have demonstrated an overall 2 to 5-fold elevated risk of neoplasms among transplanted patients compared with the general population [3–11], probably cause by the immunosuppressive condition [11]. A recent study that analyzed data from the Scientific Registry of Transplant Recipients (SRTR) in linkage with multiple cancer registries observed an increased cancer risk in solid organ (including liver, kidney, lung, and heart) transplant recipients, with an incidence of 1,375 per 100,000 person-years (standardized incidence ratios (SIRs), 2.10) [12].

Malignancies in liver transplant recipients are also rising, with incidences varying from 2% to 16% [13, 14], which lead to elevated overall mortality rates in this population [15–17]. Indeed, post-transplant de novo malignancies-related death has become one of the most significant causes in liver transplant recipients [18–21].

Most of the previous studies have focused on de novo malignancies following kidney (or heart, lung) transplantation or the total transplant population. Although, a few studies have illustrated de novo tumor burden in liver transplant recipients, their data were either from a single center or limited by the relatively small sample size [22]. In addition, the follow-up lengths and transplant times varied. This may have led to a bias in cancer estimation. Therefore, evidence based on registry databases to access de novo malignancies in recipients following liver transplantation, as well as identifying possible associated risk factors, is needed.

Previously we have assessed the spectrum of de novo malignancies following liver transplantation based on our single center experience [23]. In order to further investigate the more detailed information based on general transplant population, we analyzed data from SRTR in this study to evaluate the profile of post-transplant de novo malignancies and associated predictors in liver transplant recipients.

## Materials and Methods

This study used data from the SRTR, which includes data on all donors, wait-listed candidates, and transplant recipients in the US, submitted by the members of the Organ Procurement and Transplantation Network (OPTN), and has been described elsewhere. The Health Resources and Services Administration (HRSA), US Department of Health and Human Services, provides oversight for the activities of the OPTN and SRTR contractors [24]. Written informed consent was given by participants. The SRTR as well as the Ethical Committee at Zhejiang University reviewed and approved this study.

We identified 101,117 liver transplant patients who underwent liver transplantation from October 1987 to December 2009. The study included only adult (> = 18 years) patients. As a result, 12,081 cases were excluded, and the remaining 89,036 cases were analyzed.

There were 9,717 post-transplant malignancies recorded for 6,834 recipients, including 2,968 skin cancers, 1,895 recurring cancers and 4,854 non-skin malignancies. For the purpose of this study, we focused on non-skin de novo malignancies, which included those suffering from either post transplant lymphoproliferative disorder (PTLD), donor-related cancer or de novo solid malignancies. Of the 6,834 recipients, 3,845 suffered from 4,854 de novo non-skin malignancies, including 1,098 de novo hematological malignancies, 38 donor-related cases, and 3,718 de novo solid-organ malignancies. Of note, we excluded the recipients who suffered from skin cancers and tumor recurrence. So the occurrence of diagnosed de novo malignancies included in the following analysis did not include any skin cancers or recurred tumors, which constituted a large part of the post-transplant malignancies recorded in the database.

We divided the liver transplant recipients into two groups: those with de novo non-skin malignancies were in the malignancy group (Malig group), and the remaining were in the non-malignancy group (Non-Malig group).

For immunosuppression analysis, the following immunosuppression regimens were assessed: tacrolimus, cyclosporin, sirolimus, mycophenolate mofetil, steroids (excluding patients treated with steroids for rejection episodes), and induction therapy with an anti-CD25 antibody or with thymoglobulin. Individuals who took the specific drug at transplant discharge and remained on the same drug maintenance protocol for at least 6 months post transplant (or until death) were considered at stable maintenance immunosuppression, and were taken further to analyze the impact of this specific drug.

We then assessed data on de novo malignancies occurrence and compared the data with those of the general population to calculate the SIR and to estimate the 95% confidential interval (CI). Overall survival rates were compared between Malig and Non-Malig group, first for all the recipients, and then for male and female recipients separately. To determine the predictors for de novo malignancies in the liver transplant population, we used a logistic analysis model.

### Statistical Analysis

Numerical variables are described by means (plus the standard deviation, SD) and string variables by counts and percent. Expected malignancy cases were calculated by applying general population cancer rates (data derived from U.S. Cancer Statistics Working Group [25] and National Central Cancer Registry of China [26])to transplant recipients. SIR for each cancer type was calculated by observed counts/ expected counts. Ninety-five percent CIs for the SIR were generated using an exact method that assumed the observed counts following a Poisson distribution. Kaplan-Meier method was used to compare the overall survival. Univariate analysis was performed and those with a significant difference were taken forward for logistic regression analysis to access independent predictors of incidence of cancers. Two-sided 95% CIs are described and tests were performed at the 5% level using a two-sided approach.

## Results

### Demographics of the liver transplant population

Among the 3,845 recipients who developed de novo non-skin malignancies, 66.71% (2,565 cases) were male. The mean age at the time of transplant was 53.38. The underlying liver diseases are depicted in Table 1. The top three underlying liver diseases were HCV (977 cases, 25.41%), alcoholic liver disease (ALD) (741 cases, 19.27%) and nonalcoholic steatohepatitis (NASH) or idiopathic liver disease (411 cases, 10.69%).

**Table 1:** Overall characteristics of liver transplant recipients.

		Non- malig (N = 85,191)	Malig (N = 3,845)	P value
Transplant Year	1987–1994	15,791 (18.54%)	723 (18.80%)	0.673
	1995–1999	17,605 (20.67%)	1,125 (29.26%)	< 0.001
	2000–2004	23,349 (27.41%)	1,151 (29.93%)	0.001
	2005–2009	28,446 (33.39%)	846 (22.00%)	< 0.001
Age	18–34	7,192 (8.44%)	162 (4.21%)	< 0.001
	35–49	28,282 (33.20%)	1,042 (27.10%)	< 0.001
	50–64	42,595 (50.00%)	2,175 (56.57%)	< 0.001
	>65	7,122 (8.36%)	466 (12.12%)	< 0.001
Gender	M	53,840 (63.20%)	2,565 (66.71%)	< 0.001
Race	white	64,465 (75.67%)	3,196 (83.12%)	< 0.001
	African American or black	6,968 (8.18%)	204 (5.31%)	< 0.001
	Hispanic	9,634 (11.31%)	302 (7.85%)	< 0.001
	Asian	3,341 (3.92%)	112 (2.91%)	0.001
	others	783 (0.92%)	31 (0.81%)	0.539
Transplant Region	1	3,032 (3.56%)	207 (5.38%)	< 0.001
	2	12,314 (14.45%)	422 (10.98%)	< 0.001
	3	11,547 (13.55%)	494 (12.85%)	0.210
	4	7,204 (8.46%)	384 (9.99%)	0.001
	5	12,913 (15.16%)	454 (11.81%)	< 0.001
	6	2,487 (2.92%)	173 (4.50%)	< 0.001
	7	8,467 (9.94%)	525 (13.65%)	< 0.001
	8	6,113 (7.18%)	332 (8.63%)	0.001
	9	6,661 (7.82%)	200 (5.20%)	< 0.001
	10	7,098 (8.33%)	376 (9.78%)	0.002
	11	7,355 (8.63%)	278 (7.23%)	0.002
Blood Type	O	36,754 (43.14%)	1,631 (42.42%)	0.378
	A	33,312 (39.10%)	1,631 (42.42%)	0.012
	B	10,963 (12.87%)	439 (11.42%)	0.009
	AB	4,162 (4.89%)	194 (5.05%)	0.650
ABO_incompatible		881 (1.03%)	34 (0.88%)	0.411
BMI	< = 19.99	5,212 (6.12%)	227 (5.90%)	0.603
	20.00–24.99	23,573 (27.67%)	1,093 (28.43%)	0.311
	25.00–29.99	26,503 (31.11%)	1,207 (31.39%)	0.709
	> = 30.00	21,907 (25.72%)	991 (25.77%)	0.941
Re-transplant		7,846 (9.21%)	45 (1.17%)	< 0.001
Combined Transplant		3,973 (4.66%)	120 (3.12%)	< 0.001
Primary Diagnosis	HCV	26,680 (31.32%)	977 (25.41%)	< 0.001
	HBV	3,218 (3.78%)	128 (3.33%)	0.167
	Alcoholic liver disease	11,644 (13.67%)	741 (19.27%)	< 0.001
	Acute hepatic necrosis	5,291 (6.21%)	186 (4.84%)	0.001
	Autoimmune	2,970 (3.49%)	136 (3.54%)	0.862
	Metabolic disease	2,405 (2.82%)	103 (2.68%)	0.650
	PBC	4,728 (5.55%)	256 (6.66%)	0.004
	PSC	5,494 (6.45%)	362 (9.41%)	< 0.001
	NASH or idiopathic	10,088 (11.84%)	411 (10.69%)	0.031
	Other diagnosis	12,673 (14.88%)	545 (14.17%)	0.237
HCC		9,024 (10.59%)	506 (13.16%)	< 0.001
Immunosuppression	CellCept(Y/N)	17,031 (19.99%)/68,160 (80.01%)	819 (21.30%)/3,026 (78.70%)	0.047
	Cyclosporin(Y/N)	3,965 (4.65%)/81,226 (95.35%)	311 (8.09%)/3,534 (91.91%)	< 0.001
	Sirolimus(Y/N)	882 (1.04%)/84,309 (98.96%)	57 (1.48%)/3,788 (98.52%)	0.010
	Steroids(Y/N)	28,500 (33.45%)/56,691 (66.55%)	1,617 (42.05%)/2,228 (57.95%)	< 0.001
	Tacrolimus(Y/N)	28,539 (33.50%)/56,652 (66.50%)	1,487 (38.67%)/2,358 (61.33%)	< 0.001
	Anti_CD(Y/N)	3,605 (4.23%)/81,586 (95.77%)	164 (4.27%)/3,681 (95.73%)	0.907
	Thymoglobulin(Y/N)	1,750 (2.05%)/83,441 (97.95%)	60 (1.56%)/3,785 (98.44%)	0.038

Baseline characteristics were compared between recipients who developed de novo malignancies and those who did not. Significant differences were observed in transplant year, recipient’s age, gender, race and blood type (all p < 0.001). Recipients in the Malig group also had a higher re-transplant rate (p < 0.001) and combined transplant rate (p < 0.001). In terms of underlying diseases, recipients with HCC (p < 0.001), HCV (p < 0.001), ALD (p < 0.001), acute hepatic necrosis (p = 0.001), primary biliary cholangitis (PBC) (p = 0.004), primary sclerosing cholangitis (PSC) (p < 0.001) and NASH (p = 0.031) were significantly different between those two groups. For immunosuppression regimes, significant differences were observed for patients maintained on cellcept (p = 0.047), cyclosporine (p < 0.001), steroids (p < 0.001), sirolimus (p < 0.001), tacrolimus (p < 0.001) and thymoglobulin (p = 0.038). Introduction with anti CD 25 was similar between those two groups (p = 0.907). Detailed information is shown in Table 1.

### Spectrum of de novo malignancies

Transplant recipients had more than 11 times cancer risk compared with the general population (SIR, 11.55 [95% CI, 11.23–11.88]). The elevated cancer risk was similar for male (SIR, 10.52 [95% CI, 10.16–10.88]) and female recipients (SIR, 11.69 [95% CI, 11.12–12.27]) (Table 2). When assessing cancer risk of recipients stratified by age, overall cancer risk was elevated in recipients younger than 65 years, with SIR = 14.91 (95% CI, 12.83 to 16.99) in those aged 18 to 34 years, SIR = 6.08 (95% CI, 5.75 to 6.41) in those aged 35 to 49 years and SIR = 2.23 (95% CI, 2.15 to 2.32) in those aged 50 to 64 years respectively. In recipients older than 65 years, cancer risk decreased (SIR = 0.71; 95% CI, 0.65 to 0.76) (S1 Table). Cancer risk was also assessed by transplant regions and investigated based on cancer statistics of Chinese population reported by the National Central Cancer Registry of China (S2 and S3 Tables).

**Table 2:** Overall View of de novo cancer in liver transplant recipients.

		All	SIR	95% CI		male	SIR	95% CI		female	SIR	95% CI	
Hematologic	PTLD/lymphoma	1,041	52.90	56.12	49.69	713	47.88	51.40	44.37	328	53.75	59.57	47.94
	Leukemia	57	5.16	6.50	3.82	44	4.88	3.55	6.54	13	4.11	2.18	7.05
Donor related		38				26				12			
Solid Organ	Kaposi’s Sarcoma	19	53.35	32.29	83.11	16	35.46	20.83	57.62	3	91.94	18.39	269.68
	Brain	65	11.59	14.41	8.77	45	10.78	7.86	14.42	20	11.79	7.19	18.15
	Renal Carcinoma	121	8.71	10.26	7.16	86	7.19	8.71	5.67	35	9.66	6.71	13.45
	Carcinoma of Vulva, Perineum or Penis	32	11.23	7.65	15.83	7	15.51	6.21	31.91	25	31.92	20.69	46.99
	Carcinoma of the Uterus	41	1.89	1.35	2.56					41	5.15	3.69	6.98
	Ovarian	34	3.05	2.11	4.27					34	8.34	5.76	11.65
	Testicular	7	1.46	0.58	3.00	7	2.30	0.92	4.73				
	Esophagus	99	22.69	27.16	18.22	79	16.10	19.65	12.55	20	32.26	19.68	49.68
	Stomach	65	10.90	13.55	8.25	43	8.11	5.87	10.92	22	14.66	9.19	22.12
	Small Intestine	27	14.44	9.52	20.97	17	12.06	7.02	19.29	10	17.03	8.00	31.33
	Pancreas	128	12.08	14.17	9.99	91	11.86	14.30	9.43	37	10.80	7.59	14.89
	Larynx	67	19.29	23.92	14.67	57	14.86	18.72	11.00	10	20.43	9.60	37.59
	Tongue, Throat	170	61.59	70.85	52.33	144	55.50	64.56	46.43	26	46.87	30.65	68.50
	Thyroid	43	4.09	2.96	5.51	15	4.51	2.52	7.45	28	4.90	3.26	7.07
	Bladder	109	5.80	6.89	4.71	92	4.38	5.28	3.49	17	5.66	3.30	9.06
	Breast	235	4.00	4.51	3.49	11	13.93	6.84	24.95	224	5.63	6.36	4.89
	Prostate	316	2.34	2.60	2.09	316	3.70	4.11	3.29				
	Colorectal	313	7.61	8.45	6.77	174	5.73	6.59	4.88	139	10.60	12.36	8.83
	liver	458	77.94	85.08	70.80	352	61.18	67.57	54.79	106	95.54	113.73	77.35
	Lung	824	13.77	14.71	12.83	544	11.63	12.61	10.66	280	15.41	17.21	13.60
	Others	545				381				164			
Total		4,854	11.55	11.88	11.23	3,260	10.52	10.88	10.16	1,594	11.69	12.27	11.12

**Fig 1:**
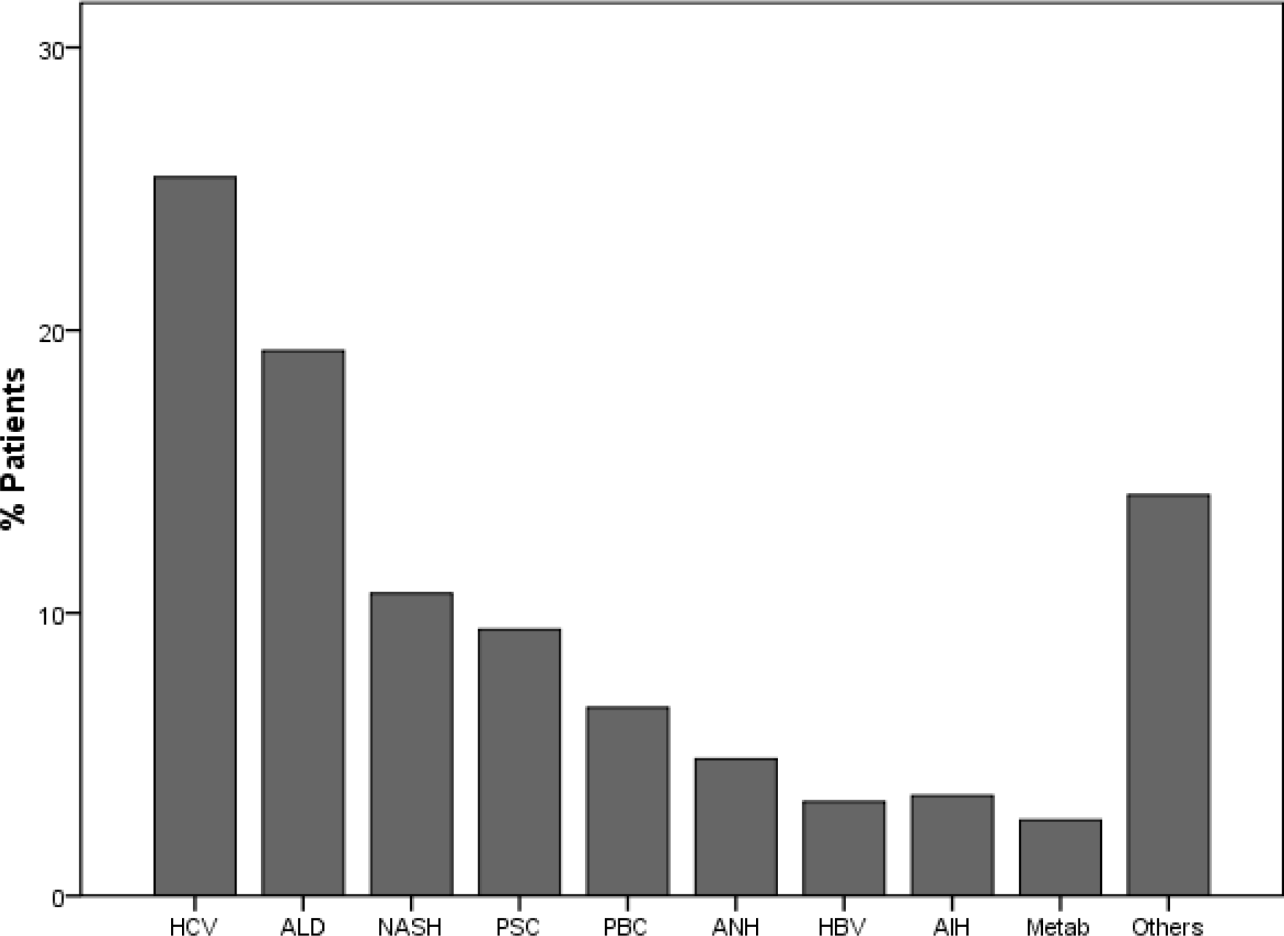
De novo malignancies in primary diagnosis. (PSC = primary sclerosing cholangitis, ALD = alcoholic liver disease, PBC = primary biliary cirrhosis, HCV = hepatitis C, NASH = cryptogenic cirrhosis, AIH = autoimmune hepatitis, AHN = acute hepatic necrosis, HBV = hepatitis B, Metab = metabolic disease.)

De novo malignancies most commonly occurred in recipients with HCV (977 (3.53% of the analyzed population) recipients developing 1,199 de novo malignancies), followed by ALD (741 cases (5.98%) developing 948 de novo malignancies) and NASH (411 cases (3.91%) developing 525 de novo malignancies) (Fig 1).

When accessing each cancer type, SIRs were elevated for both hematological cancers (PTLD/lymphoma and leukemia) and solid organ cancers (including Kaposi’s sarcoma, brain, renal carcinoma, carcinoma of vulva, perineum or penis, carcinoma of the uterus, ovarian, testicular, esophagus, stomach, small intestine, pancreas, larynx, tongue, throat, thyroid, bladder, breast, prostate, colorectal, liver and lung). Cancer risk in the liver had the highest SIR in all liver transplant recipients (SIR, 77.94 [95% CI, 70.80–85.08]), and also for male recipients (SIR, 61.18 [95% CI, 54.79–67.57]) and female recipients (SIR, 95.54 [95% CI, 77.35–113.73]).

The most common anatomical sites for developing de novo solid organ malignancies were the lung (824 observed cases), followed by the liver (458 observed cases), prostate (316 observed cases) and colon-rectal tumor (313 observed cases). In male recipients, anatomical sites for developing de novo solid organ malignancies were similar to the general population, with lung (544 observed cases), liver (352 observed cases) and prostate (316 observed cases) ranking as the three most frequent types. In female recipients, lung cancer was also the most commonly encountered type (280 observed cases), but the next most common site was the breast (224 observed cases).

### Detection of de novo malignancies during recipient follow up

The most frequent year to detect de novo malignancies during follow-up was the 2nd year post transplant, with 550 detected cases. The incidence increased in the first two years, with 315 cases in the first six months and 415 cases in the first year. The incidence then decreased gradually, and at the longest follow up time (22 years), there were only two cases.

**Fig 2:**
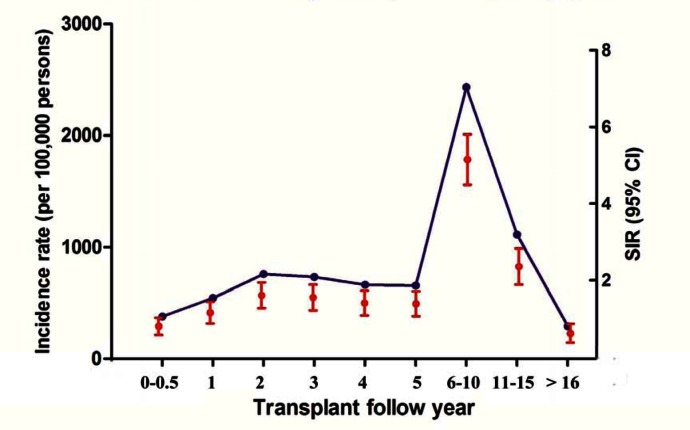
De novo malignancies by transplant follow-up year.

Compared with the general population, cancer risk increased from the first post-transplant year (SIR 1.16, 95% CI 1.04–1.27), gradually increased and reached its highest value highest years 6–10 of follow up (SIR 5.11, 95% CI 4.84–5.37). During the first 6 months post-transplant and after more than 16 years, cancer risk was decreased (SIR 0.80, 95% CI 0.71–0.89; SIR 0.62, 95% CI 0.52–0.72) (Fig 2).

### Post-transplant Survival

**Fig 3:**
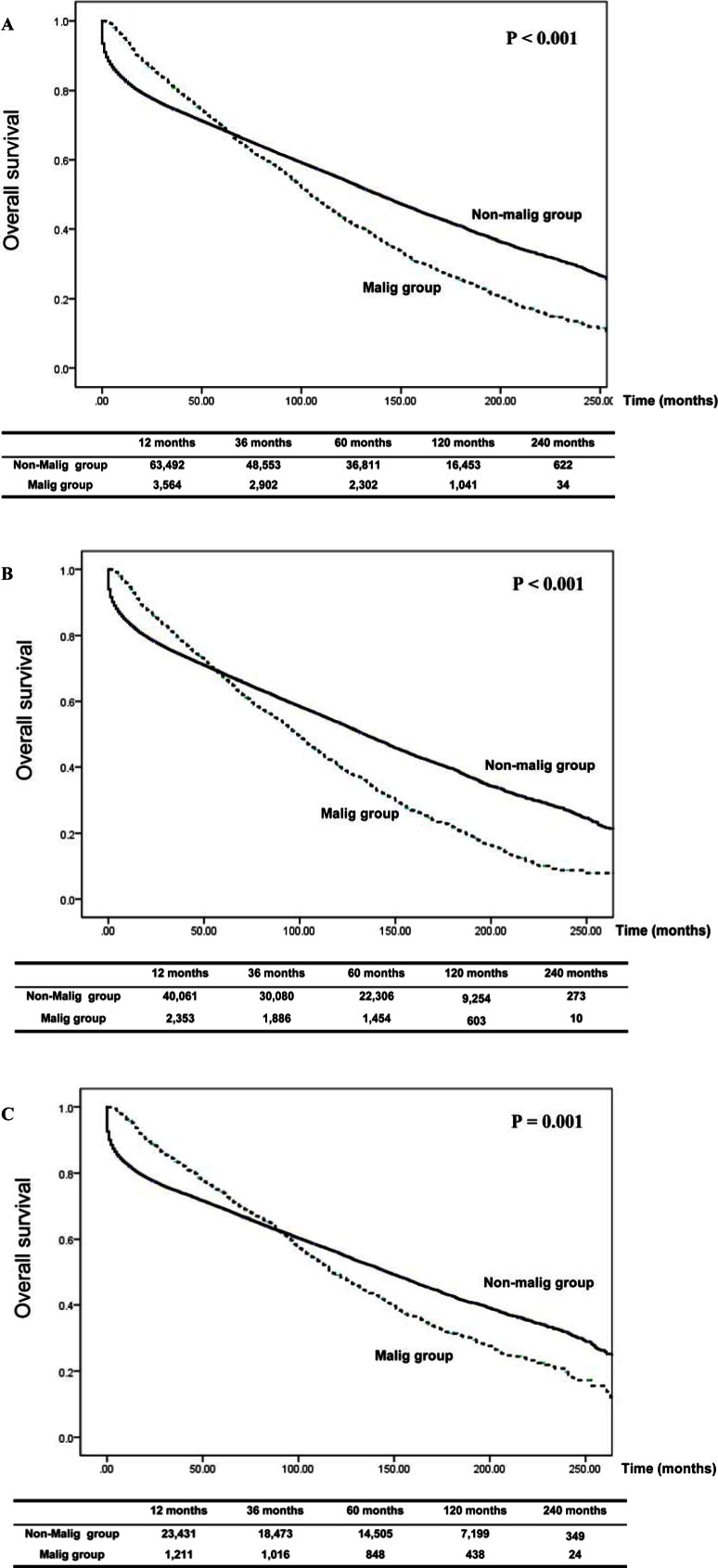
**Comparison of overall survival between:** A) Recipients with de novo cancers and those without de novo cancers; B) Male recipients with and without de novo cancers; C) Female recipients with and without de novo cancers.

For recipients with de novo malignancies, the 1-year, 3- year, 5- year and 10-year overall survival was 94.5%, 80.8%, 69.6% and 43.6%, whereas for recipients without de novo malignancies, the corresponding overall survival was 82.5%, 74.5%, 68.6% and 54.5%, respectively (p < 0.001) (Fig 3A).

We also compared the overall survival between Malig and Non-malig group for male and female recipients respectively. The 1-year, 3-year, 5-year and 10-year overall survival was 93.8%, 79.5%, 67.3%, 40.5% for male recipients with de novo malignancies, and 82.9%, 74.5%, 68.3%, 53.5% for those without de novo malignancies (p < 0.001) (Fig 3B). The corresponding 1-year, 3-year, 5-year and 10-year overall survival for female recipients with de novo malignancies was 95.9%, 83.5%, 74.2%, 49.2%, and these were 81.8%, 74.6%, 69.3%, 56.0% for female without de novo malignancies (p = 0.001) (Fig 3C).

### Risk factors for post-transplant de novo malignancies

In univariate analysis, 15 variables were associated with the development of neoplasia (S4 Table). Multivariate analysis identified the following variables as risk factors: underlying liver disease of HCV (HR 0.843, 95% CI 0.752–0.945, p = 0.003), ALD (HR 1.349, 95% CI 1.191–1.527, p < 0.001), autoimmune liver disease (HR 1.251, 95% CI 1.022–1.532, p = 0.03), NASH (HR 1.686, 95% CI 1.455–1.953, p < 0.001), re-transplantation (HR 0.128, 95% CI 0.095–0.172, p < 0.001), combined transplantation (HR 0.795, 95% CI 0.659–0.959, p = 0.016), HCC (HR 1.732, 95% CI 1.544–1.944, p < 0.001), immunosuppression regime of cellcept (HR 1.119, 95% CI 1.018–1.23, p = 0.02), cyclosporine (HR 1.513, 95% CI 1.317–1.737, p < 0.001), sirolimus (HR 1.396, 95% CI 1.061–1.838, p = 0.017), steroids (HR 1.167, 95% CI 1.081–1.26, p < 0.001) and tacrolimus (HR 1.271, 95% CI 1.168–1.383, p < 0.001) (Table 3).

**Table 3:** Logistic regression analysis.

	Sig.	OR	95% CI	
HCV *vs*. other diagnosis	0.003	0.843	0.752	0.945
Alcoholic liver disease *vs*. other diagnosis	< 0.001	1.349	1.191	1.527
Autoimmune *vs*. other diagnosis	0.03	1.251	1.022	1.532
NASH *vs*. other diagnosis	< 0.001	1.686	1.455	1.953
HCC	< 0.001	1.732	1.544	1.944
1995–1999 *vs*. 1987–1994	< 0.001	2.426	2.15	2.738
2000–2004 *vs*. 1987–1994	< 0.001	2.831	2.543	3.152
2005–2009 *vs*. 1987–1994	< 0.001	2.124	1.928	2.339
Male *vs*. Female	< 0.001	1.168	1.083	1.259
Re-transplantation	< 0.001	0.128	0.095	0.172
Combined-transplantation	0.016	0.795	0.659	0.959
35–49 *vs*. 18–34	< 0.001	0.379	0.314	0.458
50–64 *vs*. 18–34	< 0.001	0.555	0.494	0.624
> 65 *vs*. 18–34	< 0.001	0.808	0.727	0.897
CellCept	0.02	1.119	1.018	1.23
Cyclosporin	< 0.001	1.513	1.317	1.737
Sirolimus	0.017	1.396	1.061	1.838
Steroids	< 0.001	1.167	1.081	1.26
Tacrolimus	< 0.001	1.271	1.168	1.383

## Discussion

Mortality caused by post-transplant malignancies remains the leading cause of late death, significantly impairing long-term survival. Na et al. observed cancer-related mortality was significantly elevated compared with the general population (SMR = 2.83) [27]. Previous studies revealed a 2-fold increase in the rate of solid organ malignancies and a 30-fold or higher increase in the rate of PTLD. In this national registry based study, de novo malignancies occurred in 4.32% of liver transplant recipients. Cancer risk was one 10^th^ elevated in transplant recipients compared with the general population during follow-up for 23 years. This is higher than previously reported SIRs based on either single or other population based studies, and probably results from the longer follow up time and large transplant population included in the current study, which provide a more thorough estimation of cancer risk in liver transplant recipients.

For single cancer risk (after excluding skin cancers and recurred tumors), liver cancer was most elevated for both male and female recipients, which agreed with a previous study by Engels et al [12]. Thus, the high incidence of de novo liver cancer is cautionary, and intense screening after transplantation is important. In our study, all the cancers recorded had elevated SIRs. Previous studies also observed elevated GI malignancies, head and neck cancers, lung cancer, and genitourinary cancers in liver transplant recipients. Although the risks of prostate cancer and breast cancer were not reported to be elevated previously [6, 28], possibly because of intense surveillance for these cancers, our analysis still revealed a higher risk in liver transplant recipients. Thus, based on our study, a potential higher risk exists for these cancers, requiring more effective methods of intervention.

In our analysis, cancer risk was elevated in recipients less than 64 years old, and decreased by age stratification. In recipients older than 65 years old, cancer risk was lower than in the general population. This may be due to the increasing cancer incidence with age in the general population [29]. Moreover, older transplant recipients have lower post-transplant life expectancy compared with younger recipients, and may die before they suffer from a de novo malignancy. This may all contribute to the observation in our analysis.

Cancer risks also varied by follow-up year. De novo malignancy was most frequently observed in the 2^nd^ year. And the cancer risk was mostly elevated during the 6^th^ to 10^th^ years. Engels et al. found that liver cancer had extraordinary risk in the first half year following transplantation [12]. Generally, de novo solid organ cancers occur more commonly after the first transplant year and increase by age, while PTLD has its greatest incidence in the first 12 to 18 months [30]. This data may aid surveillance procedures for liver transplant recipients.

A group of baseline characteristics were significantly different between Malig and Non-Malig group, indicating a possible intrinsic difference in the oncogenesis mechanism between these two groups. Previous studies demonstrated that age, immunosuppression, environmental exposures (i.e. smoking), infection and underlying liver disease were predictors for developing non-skin solid organ malignancies in liver transplant recipients. However, those studies were limited by their relatively small sample sizes. We performed logistic regression analysis based on our national database and observed that age, transplant year, underlying liver diseases, re-transplantation and combined transplantation, and immunosuppression were associated with de novo cancer.

Alcohol as a risk factor for several cancers, including oropharyngeal, laryngeal, esophageal, and liver malignancies, is well understood, and studies have proposed several mechanisms for this carcinogenesis. Our population-based analysis also revealed that alcohol exposure was an independent predictor that increased the cancer risk following transplantation. This agrees with a previous prospective study which analyzed the National Institute of Diabetes and Digestive and Kidney Diseases Liver Transplantation Database [22]. Other underlying liver diseases associated with increased de novo cancer risk included autoimmune liver disease, NASH and HCC. Importantly, according to a recent study by Charltono et al.[31], NASH has become the 3^rd^ most common indication for liver transplantation in the United States. Although, this study demonstrated similar short survival rates for NASH compared with other liver diseases, the long-term outcome remains unknown. Thus, regarding the effects of de novo cancer on long-term survival, close monitoring during the post-transplant follow-up year in this sub-population is worth considering. Our analysis also demonstrated that recipients with HCV had the highest frequency of developing de novo cancer, yet logistic analysis revealed that HCV could reduce the risk of de novo malignancies. This may be because the transplant sample of HCV recipients was large, leading to higher detection frequency in this subgroup. Yet, among recipients with HCV, those who developed de novo cancer represented only 3.53% of all the HCV recipients, which is lower than those with ALD and NASH. This is consistent with the logistic analysis. Interestingly, re-transplantation and combined transplantation were also associated with a decreased cancer risk. This may reflect the different immune statuses in those recipients; however, the detailed mechanisms could not be determined based on the current study.

Immunosuppression regimes have long been recognized as one of the causes of post-transplant malignancies. The role of different immunosuppression protocols in transplant recipients has been explored. The mTOR inhibitors have strong anti-tumor effects compared with CNI-mediated immunosuppression in mouse models and in renal or heart transplant recipients [32–36]. However, there is limited data concerning their roles in liver transplant recipients. One study by Toso et al. revealed that sirolimus-based immunosuppression was associated with increased survival in recipients with HCC [37]. However, there is insufficient data on HCC recurrence; therefore, the antitumor/pro-tumor characteristics possibly underlying in different immunosuppression states cannot be fully defined. We found that all the immunosuppression regimes could elevate de novo cancer risk, with cyclosporin having the strongest potential effects. Based on our analysis, minimization of immunosuppression to the lowest tolerable level is recommended; however, it might not be helpful to convert the CNI regime to mTOR immunosuppression regime. Nonetheless, the benefits of reducing of skin malignancies and Kaposi’s sarcoma have been observed by immunosuppression reduction and cancer-specific treatments; however, the impact on solid tumors remains unknown. Thus, the effect of immunosuppression on de novo cancer development in the liver transplant setting requires more evidence from clinical trials and basic researches.

There are some limitations in our study. Firstly, because this was a registry-based observational study, we identified independent predictors for developing de novo malignancies, but could not fully explain the underlying mechanisms. However, this was the first large population-based study to assess the risk factors associated with de novo malignancies following transplantation, as such, it could indicate directions for basic research in carcinogenesis in the transplant setting and also act as a guide for post-transplant surveillance in clinical practice. Secondly, variables of previously identified factors, such as smoking and infectious agents as risk factors for de novo malignancies are not complete for analysis from the SRTR database; therefore, we could not determine their effects on this large population. However, the elevated cancer risk known to be associated with infection (such as cancer of the liver, stomach and Kaposi’s Sarcoma) in this study reflected the fact that infection is an important factor that correlates with de novo malignancies.

In conclusion, de novo cancer risk was more than 11 times elevated in liver transplant recipients compared with the general population. Recipient age, underlying liver diseases, re-transplantation and combined transplantation and immunosuppression were all independent predictors for developing de novo cancer. Our findings will be useful in the liver transplant decision-making process in terms of donor allocation and immunosuppression selection, as well as promoting basic researches into the carcinogenesis mechanisms in the liver transplant setting.

## Supporting Information

S1 Table De novo malignancies by age. (DOC)

S2 Table De novo malignancies by region. (DOC)

S3 Table De novo malignancies based on Chinese population. (DOC)

S4 Table Univariate analysis. (DOC)
