# Supplementary material for: Spectrum of De Novo Cancers and Predictors in Liver Transplantation: Analysis of the Scientific Registry of Transplant Recipients Database
Source: PLoS One. 2016 May 12;11(5):e0155179. doi: 10.1371/journal.pone.0155179 (PMC4865237; doi:10.1371/journal.pone.0155179)
Supplement: S1 Table — (DOC) [file pone.0155179.s001.doc]

S1 Table. De novo malignancies by age

|  |  | 18-34 | SIR | 95% CI | |  | 35-49 | SIR | 95% CI | |  | 50-64 | SIR | 95% CI | |  | >=65 | SIR | 95% CI | |
| --- | --- | --- | --- | --- | --- | --- | --- | --- | --- | --- | --- | --- | --- | --- | --- | --- | --- | --- | --- | --- |
| Hematologic |  | 7354 |  |  |  |  | 29324 |  |  |  |  | 44770 |  |  |  |  | 7588 |  |  |  |
|  | PTLD/lymphoma | 101 | 67.99 | 54.73 | 81.25 |  | 307 | 33.88 | 30.09 | 37.67 |  | 536 | 15.75 | 14.42 | 17.09 |  | 97 | 2.42 | 1.94 | 2.90 |
|  | Leukemia | 4 | 6.72 | 17.12 | 1.68 |  | 19 | 5.35 | 8.34 | 3.24 |  | 27 | 1.77 | 2.57 | 1.17 |  | 7 | 0.28 | 0.58 | 0.11 |
| Solid Organ |  |  |  |  |  |  |  |  |  |  |  |  |  |  |  |  |  |  |  |  |
|  | Kaposi’s Sarcoma |  |  |  |  |  | 3 | 3.93 | 11.54 | 0.79 |  | 11 | 24.57 | 10.05 | 39.09 |  | 5 | 14.32 | 1.77 | 26.88 |
|  | Brain | 3 | 4.64 | 13.60 | 0.93 |  | 15 | 3.55 | 5.87 | 1.99 |  | 37 | 2.52 | 3.47 | 1.77 |  | 10 | 1.30 | 2.38 | 0.61 |
|  | Renal Carcinoma | 4 | 13.27 | 33.83 | 3.32 |  | 62 | 7.42 | 5.57 | 9.27 |  | 47 | 1.04 | 1.39 | 0.77 |  | 8 | 0.33 | 0.65 | 0.14 |
|  | Ovarian | 1 | 1.92 | 10.73 | 0.19 |  | 10 | 1.21 | 2.23 | 0.57 |  | 18 | 0.50 | 0.79 | 0.30 |  | 5 | 0.28 | 0.66 | 0.09 |
|  | Esophagus | 2 | 135.98 | 489.53 | 13.60 |  | 21 | 15.24 | 23.22 | 9.43 |  | 67 | 4.65 | 3.53 | 5.76 |  | 9 | 0.97 | 1.84 | 0.43 |
|  | Stomach | 3 | 27.20 | 79.78 | 5.44 |  | 13 | 5.04 | 8.64 | 2.67 |  | 38 | 2.59 | 3.55 | 1.83 |  | 11 | 0.78 | 1.40 | 0.38 |
|  | Pancreas | 4 | 67.99 | 173.38 | 17.00 |  | 30 | 10.77 | 15.36 | 7.25 |  | 75 | 2.78 | 2.15 | 3.41 |  | 19 | 0.71 | 1.10 | 0.43 |
|  | Larynx | 0 | 0.00 | 0.00 | 0.00 |  | 16 | 10.91 | 17.73 | 6.41 |  | 43 | 3.22 | 4.34 | 2.33 |  | 8 | 1.35 | 2.67 | 0.57 |
|  | Thyroid | 5 | 2.55 | 5.96 | 0.81 |  | 16 | 1.03 | 1.68 | 0.61 |  | 20 | 0.70 | 1.09 | 0.43 |  | 2 | 0.29 | 1.05 | 0.03 |
|  | Prostate |  |  |  |  |  | 28 | 1.68 | 2.42 | 1.11 |  | 216 | 0.46 | 0.40 | 0.52 |  | 72 | 0.25 | 0.19 | 0.31 |
|  | Colorectal | 15 | 26.84 | 44.37 | 15.03 |  | 92 | 5.57 | 4.43 | 6.71 |  | 166 | 1.55 | 1.31 | 1.78 |  | 40 | 0.41 | 0.56 | 0.30 |
|  | liver | 11 | 249.30 | 446.47 | 122.38 |  | 141 | 155.11 | 129.51 | 180.71 |  | 242 | 14.22 | 12.43 | 16.02 |  | 64 | 5.56 | 4.20 | 6.92 |
|  | Lung | 6 | 74.17 | 161.94 | 27.20 |  | 209 | 43.73 | 37.80 | 49.65 |  | 508 | 5.84 | 5.33 | 6.35 |  | 101 | 0.64 | 0.52 | 0.77 |
| Total |  | 197 | 14.91 | 12.83 | 16.99 |  | 1292 | 6.08 | 5.75 | 6.41 |  | 2743 | 2.23 | 2.15 | 2.32 |  | 584 | 0.71 | 0.65 | 0.76 |
