# Supplementary material for: Spectrum of De Novo Cancers and Predictors in Liver Transplantation: Analysis of the Scientific Registry of Transplant Recipients Database
Source: PLoS One. 2016 May 12;11(5):e0155179. doi: 10.1371/journal.pone.0155179 (PMC4865237; doi:10.1371/journal.pone.0155179)
Supplement: S2 Table — (DOC) [file pone.0155179.s002.doc]

S2 Table. De novo malignancies by region

|  |  | All | region 1 | | | | region 2 | | | | region 3 | | | | region 4 | | | | region 5 | | | |
| --- | --- | --- | --- | --- | --- | --- | --- | --- | --- | --- | --- | --- | --- | --- | --- | --- | --- | --- | --- | --- | --- | --- |
|  |  | Cases | Cases | SIR | 95%CI | | Cases | SIR | 95%CI | | Cases | SIR | 95%CI | | Cases | SIR | 95%CI | | Cases | SIR | 95%CI | |
| Hematologic | PTLD/lymphoma | 1041 | 55 | 71.65 | 90.58 | 52.71 | 135 | 47.32 | 55.30 | 39.34 | 136 | 55.19 | 64.46 | 45.91 | 97 | 61.31 | 73.51 | 49.11 | 141 | 53.34 | 62.15 | 44.54 |
|  | Leukemia | 57 | 2 | 4.47 | 0.45 | 16.11 | 3 | 1.79 | 0.36 | 5.25 | 2 | 1.37 | 0.14 | 4.93 | 6 | 5.97 | 2.19 | 13.03 | 10 | 6.02 | 2.83 | 11.08 |
| Donor related | | 38 | 3 |  |  |  | 5 |  |  |  | 2 |  |  |  | 3 |  |  |  | 8 |  |  |  |
| Solid Organ | Kaposi’s Sarcoma | 19 | 0 |  |  |  | 2 | 28.55 | 2.86 | 102.79 | 2 | 39.86 | 3.99 | 143.51 | 4 | 175.72 | 43.93 | 448.08 | 3 | 64.12 | 12.82 | 188.10 |
|  | Brain | 65 | 3 | 13.23 | 2.65 | 38.81 | 6 | 7.01 | 2.57 | 15.31 | 10 | 13.01 | 6.11 | 23.94 | 1 | 2.04 | 0.20 | 11.44 | 10 | 11.83 | 5.56 | 21.76 |
|  | Renal Carcinoma | 121 | 1 | 1.98 | 0.20 | 11.08 | 16 | 7.81 | 4.59 | 12.69 | 6 | 2.92 | 1.07 | 6.37 | 26 | 19.14 | 12.52 | 27.98 | 12 | 6.34 | 3.28 | 11.10 |
|  | Carcinoma of the Uterus | 41 | 1 | 1.02 | 0.10 | 5.71 | 6 | 1.60 | 0.59 | 3.50 | 8 | 3.31 | 1.40 | 6.53 | 5 | 3.21 | 1.03 | 7.52 | 3 | 0.96 | 0.19 | 2.83 |
|  | Ovarian | 34 | 0 |  |  |  | 2 | 1.33 | 0.13 | 4.78 | 11 | 8.41 | 4.13 | 15.06 | 6 | 6.91 | 2.53 | 15.08 | 3 | 1.91 | 0.38 | 5.60 |
|  | Testicular | 7 | 0 |  |  |  | 1 | 1.44 | 0.14 | 8.04 | 0 |  |  |  | 1 | 2.66 | 0.27 | 14.91 | 0 |  |  |  |
|  | Esophagus | 99 | 12 | 62.79 | 32.44 | 109.89 | 13 | 20.35 | 10.80 | 34.90 | 18 | 32.15 | 19.11 | 50.72 | 7 | 21.45 | 8.58 | 44.13 | 9 | 17.72 | 7.87 | 33.66 |
|  | Stomach | 65 | 2 | 9.22 | 0.92 | 33.18 | 3 | 3.30 | 0.66 | 9.69 | 10 | 12.65 | 5.94 | 23.27 | 7 | 15.38 | 6.15 | 31.63 | 10 | 12.31 | 5.79 | 22.66 |
|  | Pancreas | 128 | 7 | 16.88 | 6.75 | 34.73 | 11 | 6.54 | 3.21 | 11.72 | 19 | 12.59 | 7.62 | 19.61 | 11 | 12.34 | 6.06 | 22.10 | 11 | 7.45 | 3.66 | 13.34 |
|  | Larynx | 67 | 4 | 33.38 | 8.34 | 85.11 | 16 | 31.41 | 18.45 | 51.04 | 6 | 10.60 | 3.89 | 23.15 | 6 | 21.96 | 8.05 | 47.96 | 1 | 3.40 | 0.34 | 19.04 |
|  | Tongue, Throat | 170 | 5 | 13.42 | 4.30 | 31.41 | 22 | 15.29 | 9.59 | 23.07 | 22 | 14.42 | 9.05 | 21.77 | 13 | 14.46 | 7.67 | 24.80 | 14 | 11.35 | 6.24 | 19.06 |
|  | Thyroid | 43 | 6 | 10.18 | 3.73 | 22.22 | 2 | 0.99 | 0.10 | 3.57 | 0 |  |  |  | 4 | 4.73 | 1.18 | 12.06 | 6 | 3.01 | 1.10 | 6.57 |
|  | Bladder | 109 | 7 | 8.25 | 3.30 | 16.97 | 19 | 6.77 | 4.10 | 10.54 | 11 | 4.84 | 2.38 | 8.67 | 8 | 6.01 | 2.55 | 11.86 | 8 | 3.45 | 1.47 | 6.82 |
|  | Breast | 235 | 7 | 1.61 | 0.64 | 3.31 | 15 | 0.92 | 0.52 | 1.52 | 39 | 2.76 | 1.96 | 3.77 | 25 | 2.84 | 1.84 | 4.18 | 26 | 1.70 | 1.11 | 2.48 |
|  | Prostate | 316 | 20 | 4.59 | 2.80 | 7.08 | 51 | 2.71 | 3.45 | 1.97 | 47 | 2.71 | 1.99 | 3.61 | 18 | 1.94 | 1.15 | 3.06 | 41 | 2.54 | 1.82 | 3.44 |
|  | Colorectal | 313 | 20 | 15.21 | 9.28 | 23.42 | 30 | 5.44 | 3.66 | 7.76 | 44 | 8.09 | 5.89 | 10.85 | 33 | 10.33 | 7.11 | 14.49 | 44 | 9.14 | 6.65 | 12.26 |
|  | liver | 458 | 26 | 113.06 | 73.92 | 165.24 | 48 | 48.01 | 35.31 | 63.61 | 49 | 59.41 | 43.77 | 78.56 | 54 | 83.23 | 105.43 | 61.03 | 79 | 77.26 | 94.29 | 60.22 |
|  | Lung | 824 | 47 | 21.40 | 15.71 | 28.46 | 109 | 12.77 | 15.16 | 10.37 | 120 | 13.77 | 16.23 | 11.30 | 77 | 15.46 | 18.91 | 12.00 | 88 | 15.32 | 18.52 | 12.12 |
|  | other |  | 29 |  |  |  | 41 |  |  |  | 90 |  |  |  | 37 |  |  |  | 63 |  |  |  |
| Total |  | 4854 | 264 | 16.57 | 14.57 | 18.57 | 564 | 9.09 | 9.84 | 8.34 | 659 | 11.73 | 12.63 | 10.84 | 456 | 13.59 | 14.84 | 12.34 | 596 | 10.89 | 11.77 | 10.02 |

**To be continued**

**continued**

|  |  | region 6 | | | | region 7 | | | | region 8 | | | | region 9 | | | | region 10 | | | | region 11 | | | |
| --- | --- | --- | --- | --- | --- | --- | --- | --- | --- | --- | --- | --- | --- | --- | --- | --- | --- | --- | --- | --- | --- | --- | --- | --- | --- |
|  |  | Cases | SIR | 95%CI | | Cases | SIR | 95%CI | | Cases | SIR | 95%CI | | Cases | SIR | 95%CI | | Cases | SIR | 95%CI | | Cases | SIR | 95%CI | |
| Hematologic | PTLD/lymphoma | 61 | 106.66 | 133.43 | 79.89 | 147 | 68.80 | 79.93 | 57.68 | 101 | 71.02 | 84.87 | 57.17 | 42 | 24.39 | 31.76 | 17.01 | 74 | 44.07 | 54.11 | 34.03 | 52 | 32.53 | 41.38 | 23.69 |
|  | Leukemia | 4 | 11.25 | 2.81 | 28.69 | 15 | 11.12 | 6.23 | 18.39 | 2 | 2.23 | 0.22 | 8.03 | 1 | 0.96 | 0.10 | 5.37 | 9 | 9.43 | 4.19 | 17.92 | 3 | 3.07 | 0.61 | 8.99 |
|  | Donor related | 1 |  |  |  | 4 |  |  |  | 2 |  |  |  | 4 |  |  |  | 4 |  |  |  | 2 |  |  |  |
| Solid Organ | Kaposi’s Sarcoma | 0 |  |  |  | 2 | 111.21 | 11.12 | 400.36 | 1 | 93.10 | 9.31 | 521.33 | 1 | 18.22 | 1.82 | 102.03 | 1 | 57.34 | 5.73 | 321.11 | 3 | 196.52 | 39.30 | 576.44 |
|  | Brain | 4 | 22.06 | 5.52 | 56.25 | 6 | 9.93 | 3.64 | 21.68 | 8 | 17.40 | 7.40 | 34.37 | 7 | 15.46 | 6.18 | 31.80 | 5 | 9.65 | 2.51 | 22.58 | 5 | 9.72 | 2.53 | 22.74 |
|  | Renal Carcinoma | 8 | 20.51 | 8.71 | 40.50 | 24 | 16.56 | 10.62 | 24.56 | 11 | 10.68 | 5.24 | 19.12 | 2 | 1.82 | 0.18 | 6.56 | 10 | 8.01 | 3.77 | 14.74 | 5 | 3.94 | 1.02 | 9.21 |
|  | Carcinoma of the Uterus | 2 | 2.88 | 0.29 | 10.35 | 6 | 2.43 | 0.89 | 5.31 | 4 | 2.47 | 0.62 | 6.31 | 1 | 0.48 | 0.05 | 2.68 | 1 | 0.49 | 0.05 | 2.72 | 4 | 2.28 | 0.57 | 5.80 |
|  | Ovarian | 0 |  |  |  | 5 | 4.95 | 1.58 | 11.58 | 3 | 4.02 | 0.80 | 11.79 | 0 |  |  |  | 1 | 1.11 | 0.11 | 6.24 | 3 | 3.52 | 0.70 | 10.33 |
|  | Testicular | 0 |  |  |  | 1 | 1.77 | 0.18 | 9.89 | 1 | 2.56 | 0.26 | 14.32 | 2 | 4.86 | 0.49 | 17.49 | 0 |  |  |  | 1 | 2.74 | 0.27 | 15.35 |
|  | Esophagus | 3 | 23.17 | 4.63 | 67.98 | 11 | 24.37 | 11.96 | 43.64 | 6 | 19.74 | 7.24 | 43.09 | 7 | 21.26 | 8.50 | 43.73 | 4 | 9.85 | 2.46 | 25.12 | 9 | 25.09 | 11.15 | 47.67 |
|  | Stomach | 2 | 11.63 | 1.16 | 41.86 | 10 | 18.53 | 8.71 | 34.10 | 11 | 32.72 | 16.06 | 58.59 | 4 | 6.78 | 1.69 | 17.29 | 5 | 11.15 | 2.90 | 26.09 | 1 | 2.09 | 0.21 | 11.68 |
|  | Pancreas | 5 | 15.53 | 4.97 | 36.35 | 17 | 15.89 | 9.25 | 25.42 | 9 | 12.36 | 5.49 | 23.48 | 7 | 7.39 | 2.96 | 15.21 | 14 | 15.03 | 8.26 | 25.22 | 17 | 18.47 | 10.75 | 29.55 |
|  | Larynx | 3 | 42.56 | 8.51 | 124.84 | 6 | 19.51 | 7.15 | 42.60 | 7 | 31.33 | 12.53 | 64.45 | 4 | 16.19 | 4.05 | 41.30 | 13 | 41.74 | 22.16 | 71.61 | 1 | 2.84 | 0.28 | 15.88 |
|  | Tongue, Throat | 7 | 22.56 | 9.02 | 46.40 | 23 | 22.28 | 14.14 | 33.32 | 22 | 30.89 | 19.38 | 46.62 | 6 | 8.33 | 3.05 | 18.18 | 19 | 22.70 | 13.74 | 35.36 | 17 | 18.44 | 10.74 | 29.50 |
|  | Thyroid | 0 |  |  |  | 13 | 11.16 | 5.92 | 19.14 | 5 | 5.57 | 1.45 | 13.04 | 1 | 0.80 | 0.08 | 4.48 | 5 | 5.44 | 1.41 | 12.73 | 1 | 1.05 | 0.11 | 5.90 |
|  | Bladder | 2 | 3.57 | 0.36 | 12.86 | 19 | 9.68 | 5.86 | 15.09 | 14 | 10.32 | 5.68 | 17.32 | 5 | 3.09 | 0.80 | 7.23 | 9 | 5.51 | 2.45 | 10.46 | 7 | 4.56 | 1.82 | 9.38 |
|  | Breast | 9 | 2.66 | 1.18 | 5.06 | 28 | 2.47 | 1.64 | 3.56 | 40 | 5.10 | 3.65 | 6.95 | 16 | 1.81 | 1.07 | 2.95 | 15 | 1.67 | 0.93 | 2.76 | 15 | 1.59 | 0.89 | 2.62 |
|  | Prostate | 17 | 5.11 | 2.98 | 8.18 | 32 | 2.60 | 1.77 | 3.67 | 24 | 2.91 | 1.86 | 4.31 | 20 | 1.90 | 1.16 | 2.92 | 23 | 2.41 | 1.53 | 3.60 | 23 | 2.28 | 1.45 | 3.41 |
|  | Colorectal | 17 | 15.51 | 9.03 | 24.81 | 42 | 10.52 | 7.59 | 14.23 | 34 | 12.38 | 8.56 | 17.30 | 9 | 3.04 | 1.35 | 5.78 | 21 | 6.54 | 4.05 | 9.97 | 19 | 5.83 | 3.53 | 9.09 |
|  | liver | 17 | 86.17 | 50.18 | 137.87 | 36 | 77.59 | 54.10 | 107.33 | 40 | 104.31 | 74.58 | 142.12 | 22 | 38.63 | 24.23 | 58.30 | 52 | 113.44 | 144.27 | 82.60 | 35 | 69.48 | 48.24 | 96.67 |
|  | Lung | 26 | 16.87 | 11.03 | 24.66 | 75 | 13.93 | 17.09 | 10.78 | 75 | 19.33 | 23.70 | 14.95 | 41 | 9.50 | 6.81 | 12.88 | 101 | 18.78 | 22.44 | 15.12 | 65 | 11.21 | 13.94 | 8.49 |
|  | other | 12 |  |  |  | 91 |  |  |  | 23 |  |  |  | 32 |  |  |  | 82 |  |  |  | 45 |  |  |  |
| Total |  | ## | 17.08 | 19.42 | 14.75 | 619 | 14.76 | 15.92 | 13.59 | 449 | 15.34 | 16.76 | 13.92 | 235 | 6.87 | 7.75 | 5.99 | 469 | 13.45 | 14.67 | 12.23 | 337 | 9.34 | 10.34 | 8.35 |
