# Supplementary material for: Spectrum of De Novo Cancers and Predictors in Liver Transplantation: Analysis of the Scientific Registry of Transplant Recipients Database
Source: PLoS One. 2016 May 12;11(5):e0155179. doi: 10.1371/journal.pone.0155179 (PMC4865237; doi:10.1371/journal.pone.0155179)
Supplement: S3 Table — (DOC) [file pone.0155179.s003.doc]

S3 Table. De novo malignancies based on Chinese population

|  |  | All | Incidence rate* | SIR | 95% CI | |  | Male | Incidence rate* | SIR | 95% CI | |  | Female | Incidence rate* | SIR | 95% CI | |
| --- | --- | --- | --- | --- | --- | --- | --- | --- | --- | --- | --- | --- | --- | --- | --- | --- | --- | --- |
| Hematologic | PTLD/lymphoma | 1041 | 6.68 | 175.03 | 185.66 | 164.40 |  | 713 | 7.71 | 163.95 | 175.99 | 151.92 |  | 328 |  |  |  |  |
| Solid Organ | Renal Carcinoma | 121 |  |  |  |  |  | 86 | 7.07 | 21.57 | 26.12 | 17.01 |  | 35 |  |  |  |  |
|  | Carcinoma of the Uterus | 41 |  |  |  |  |  |  |  |  |  |  |  | 41 | 8.77 | 14.33 | 10.27 | 19.43 |
|  | Ovarian | 34 |  |  |  |  |  |  |  |  |  |  |  | 34 | 7.95 | 13.11 | 9.06 | 18.31 |
|  | Esophagus | 99 | 22.14 | 5.02 | 6.01 | 4.03 |  | 79 | 30.44 | 4.60 | 5.62 | 3.59 |  | 20 | 13.64 | 4.49 | 2.74 | 6.92 |
|  | Stomach | 65 | 36.21 | 2.02 | 2.51 | 1.53 |  | 43 | 49.61 | 1.54 | 1.11 | 2.07 |  | 22 | 22.50 | 3.00 | 1.88 | 4.52 |
|  | Pancreas | 128 | 7.28 | 19.75 | 23.17 | 16.33 |  | 91 | 8.24 | 19.58 | 23.60 | 15.56 |  | 37 |  |  |  |  |
|  | Thyroid | 43 | 6.56 | 7.36 | 5.32 | 9.91 |  | 15 |  |  |  |  |  | 28 | 10.09 | 8.50 | 5.65 | 12.27 |
|  | Bladder | 109 | 6.61 | 18.52 | 22.00 | 15.04 |  | 92 | 9.78 | 16.68 | 20.09 | 13.27 |  | 17 |  |  |  |  |
|  | Breast | 235 | 21.21 | 12.44 | 14.04 | 10.85 |  | 11 |  |  |  |  |  | 224 | 42.55 | 16.13 | 18.25 | 14.02 |
|  | Prostate | 316 |  |  |  |  |  | 316 | 9.92 | 56.48 | 62.70 | 50.25 |  |  |  |  |  |  |
|  | Colo-rectal | 313 | 29.44 | 11.94 | 13.26 | 10.62 |  | 174 | 32.38 | 9.53 | 10.94 | 8.11 |  | 139 | 26.42 | 16.12 | 18.80 | 13.44 |
|  | liver | 458 | 28.71 | 17.92 | 19.56 | 16.28 |  | 352 | 41.99 | 14.86 | 16.41 | 13.31 |  | 106 | 15.11 | 21.50 | 25.59 | 17.41 |
|  | Lung | 824 | 53.57 | 17.28 | 18.46 | 16.10 |  | 544 | 70.40 | 13.70 | 14.85 | 12.55 |  | 280 | 36.34 | 23.61 | 26.38 | 20.85 |
|  |  |  |  |  |  |  |  |  |  |  |  |  |  |  |  |  |  |  |
| Total |  | 4854 | 285.91 | 19.07 | 19.60 | 18.53 |  | 317.97 | 18.18 | 18.80 | 17.55 |  |  | 1594 | 253.09 | 19.30 | 20.25 | 18.35 |

*Data from National Central Cancer Registry of China

**Reference:**

1. Chen W, Zheng R, Zhang S, et al. Report of incidence and mortality in China cancer registries, 2009. Chin J Cancer Res 2013; 25(1):10-21.
