# Supplementary material for: Spectrum of De Novo Cancers and Predictors in Liver Transplantation: Analysis of the Scientific Registry of Transplant Recipients Database
Source: PLoS One. 2016 May 12;11(5):e0155179. doi: 10.1371/journal.pone.0155179 (PMC4865237; doi:10.1371/journal.pone.0155179)
Supplement: S4 Table — (DOC) [file pone.0155179.s004.doc]

S4 Table. Univariate analysis

|  | Sig. | OR | 95% CI | |
| --- | --- | --- | --- | --- |
|  |  |  | Lower | Upper |
| Other Races | < 0.001 |  |  |  |
| White | 0.222 | 1.252 | 0.873 | 1.796 |
| African American or Black | 0.124 | 0.739 | 0.503 | 1.087 |
| Hispanic | 0.225 | 0.792 | 0.543 | 1.154 |
| Asian | 0.421 | 0.847 | 0.565 | 1.27 |
| Other diagnosis | < 0.001 |  |  |  |
| HCV | 0.003 | 0.852 | 0.765 | 0.948 |
| HBV | 0.436 | 0.925 | 0.76 | 1.126 |
| Alcoholic liver disease | < 0.001 | 1.48 | 1.321 | 1.658 |
| AHN | 0.02 | 0.817 | 0.69 | 0.968 |
| Autoimmune | 0.522 | 1.065 | 0.879 | 1.29 |
| Metabolic disease | 0.97 | 0.996 | 0.803 | 1.235 |
| PBC | 0.003 | 1.259 | 1.081 | 1.466 |
| PSC | < 0.001 | 1.532 | 1.337 | 1.756 |
| NASH | 0.417 | 0.947 | 0.831 | 1.08 |
| Transplant year 1987-1994 | < 0.001 |  |  |  |
| 1995-1999 | < 0.001 | 1.539 | 1.391 | 1.703 |
| 2000-2004 | < 0.001 | 2.149 | 1.961 | 2.354 |
| 2005-2009 | < 0.001 | 1.658 | 1.514 | 1.814 |
| Gender | < 0.001 | 1.167 | 1.09 | 1.25 |
| ABO incompatible | 0.368 | 0.854 | 0.605 | 1.204 |
| Re transplantation | < 0.001 | 0.117 | 0.087 | 0.157 |
| Combined transplantation | < 0.001 | 0.659 | 0.548 | 0.792 |
| Age at transplant 18-34 | < 0.001 |  |  |  |
| 35-49 | < 0.001 | 0.344 | 0.287 | 0.413 |
| 50-64 | < 0.001 | 0.563 | 0.503 | 0.63 |
| > 65 | < 0.001 | 0.780 | 0.704 | 0.865 |
| Blood type AB | 0.015 |  |  |  |
| O | 0.527 | 0.952 | 0.818 | 1.109 |
| A | 0.817 | 1.018 | 0.874 | 1.186 |
| B | 0.085 | 0.859 | 0.723 | 1.021 |
| CellCept | 0.047 | 1.083 | 1.001 | 1.172 |
| Cyclosporin | < 0.001 | 1.803 | 1.599 | 2.033 |
| Sirolimus | 0.008 | 1.438 | 1.098 | 1.884 |
| Steroids | < 0.001 | 1.444 | 1.352 | 1.542 |
| Tacrolimus | < 0.001 | 1.252 | 1.171 | 1.338 |
| Thymoglobulin | 0.034 | 0.756 | 0.583 | 0.98 |
| HCC | < 0.001 | 1.279 | 1.162 | 1.408 |
